# Effect of Surface Densification on the Microstructure and Mechanical Properties of Powder Metallurgical Gears by Using a Surface Rolling Process

**DOI:** 10.3390/ma9100846

**Published:** 2016-10-19

**Authors:** Jingguang Peng, Yan Zhao, Di Chen, Kiade Li, Wei Lu, Biao Yan

**Affiliations:** 1 School of Materials Science and Engineering, Tongji University, Shanghai 201804, China; jingguangp@shautopm.com.cn (J.P.); luwis_1981@hotmail.com (Y.Z.); 2 Shanghai Automotive Powder Metallurgy Co. Ltd., Shanghai 201908, China; chendi1201@126.com (D.C.); lidekaimilan@163.com (K.L.)

**Keywords:** powder metallurgy, rolling, surface densification, wear resistance

## Abstract

Powder metallurgy (PM) components are widely used in the auto industry due to the advantage of net-shape forming, low cost, and high efficiency. Still, usage of PM components is limited in the auto industry when encountering rigorous situations, like heavy load, due to lower strength, hardness, wear resistance, and other properties compared to wrought components due to the existence of massive pores in the PM components. In this study, through combining the powder metallurgy process and rolling process, the pores in the PM components were decreased and a homogenous densified layer was formed on the surface, which resulted in the enhancement of the strength, hardness, wear resistance, and other properties, which can expand its range of application. In this paper, we study the impact of different rolling feeds on the performance of the components’ surfaces. We found that with the increase of the rolling feed, the depth of the densified layer increased.

## 1. Introduction

With the rapid development of the auto industry, new engine gears with low cost and high performance were required. Traditional machining processes of fabricating engine gears wastes massive raw material and requires expensive equipment and complex procedures, which increases the cost. Under these circumstances, gears requiring significant procedures with complex shapes are prepared with powder metallurgy (PM) methods [1,2,3]. PM methods can not only improve the production efficiency, but also dramatically decrease the production cost [4,5,6]. However, since the Fe-base components prepared with traditional PM methods also had a porosity about 5%–15%, its usable range in the auto industry was limited due to the decreased wear resistance, strength, and hardness caused by the pores [7]. Mechanical properties of Fe-base components can be comparable, even superior, to the wrought steel when the porosity is close to 0%, which requires new technologies to prepare PM components with low porosities and expand the usage in the auto industry.

Many surface modification technologies have been applied to improve the surface properties of components via a functional surface layer [8]. Bach proposed a plasma spraying technique and Kuroda studied the thermal spraying method [8]. However, long processing times and high costs are the disadvantages of these methods. Theisen developed a new ring rolling technology. Mähler and Kebriaei performed several numerical analyses on ring rolling technology [8]. To reduce the porosity in the PM components and improve their mechanical properties, different techniques, like warm compaction [9], high speed pressing [10], copper-impregnation [11], double-press and double-sinter [12], powder forging [13] and hot isostatic pressing [14], were developed to manufacture high-performance PM components. These techniques can improve PM components’ properties and more PM components can be used in the auto industry.

In many applications, the load can cause high stress at or near the surface of the part, and the stress would rapidly decline toward the core of the parts [7]. This means it is important to achieve densification in the surface. A number of studies focused on the surface densification and some researchers have successfully prepared PM components with a densified layer with a depth of about 0.1–0.2 mm, which can ensure good mechanical properties [15,16,17].

Rolling densification is an important technique among the selective densification techniques [17]. By adding stress on the workpieces through rolling, textures and structures in the surface layer can be changed via the plastic deformation of the workpieces under room temperature, which can improve the physical and mechanical properties of the workpieces. Therefore, this technique is widely used to achieve high surface fineness and better properties.

In this study, we prepared high-performance PM components by combining the PM technique and the surface densification technique. To study the impact of different rolling feeds on the performance of the PM components, density, hardness, roughness, and wear resistance of the components were tested, and scanning electron microscope (SEM) images of microstructures of densified and non-densified areas were taken to make comparisons. This study should be helpful in fabricating surface-densified components using rolling techniques.

## 2. Experimental

### 2.1. Preparation of PM Components

The raw material of the surface densified gears was pre-alloyed Hoganas powder. Steel powder was pre-alloyed powder with 0.85% Cr and mixed with 0.80% C and 1.50% Cu in Hoganas Starmix (Shanghai, China). Table 1 shows the chemical composition of PM material. 

The gears were suppressed on a mechanical press under the pressure of 450 kN as indicated in the pressure sensor. The sintering process was performed via a single normal-temperature sintering (1357 K × 30 min) route in a continuous furnace under an ammonia-dissolving atmosphere. The carbon potential was controlled so that the carbon content of the gears stayed the same as the original ones. The density of the sintered sprocket was about 7.10 g/cm^3^, which was measured by Archimedes’ method.

### 2.2. Surface Rolling Apparatus and Methods

**Figure 1:**
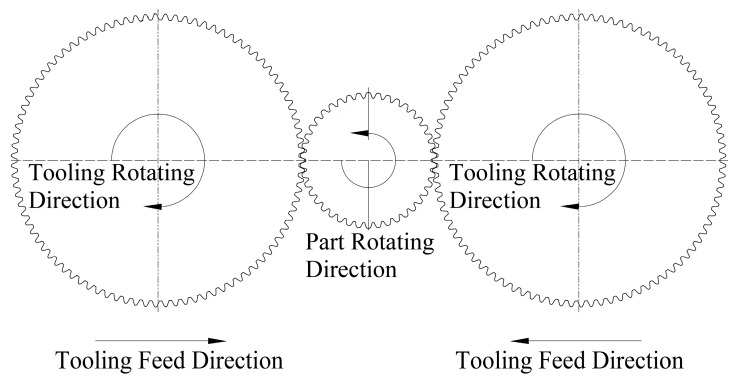
Schematic view of the experimental apparatus.

The rolling densification process was processed with a FLEX M20 HCN rolling machine from Escofier (Paris, France), showed in Figure 1. During the rolling process, two rolling dies were spinning in the same direction and the same speed. Through moving both roller-dies in the same direction, pressure on the components could be adjusted by changing the wheelbase. During the process, the rolling speed was 30 r/min and the feeding speed of the roller dies was 0.6 mm/s. After reaching the preset distance, the roller dies stopped the feed and maintained the distance for 1 s. The rolling feed △d were set at 0, 0.2, 0.4, 0.6, and 0.8 mm, separately.

Metallographs of the surface densified layer were taken using an optical microscope (Olympus, bx51, Tokyo, Japan) and more than ten images are used to estimate the depth of the surface densified layer. Vickers hardness was tested using a Vickers hardness testing machine (DHV-1000 Shanghai, Shanghai, China) for ten samples. Tooth flank roughness tests were carried with a hardness testing machine (Mitutoyo, SJ-410, Tokyo, Japan) for ten samples. For the wear resistance tests, a nano-scratch instrument (CSM, Zuric, Switzerland) was used. SEM (Apollo300, Cam Scan Ltd., Cambridge, UK) images were taken to observe the fracture morphology.

## 3. Results and Discussion

### 3.1. Microstructure of the Surface Densified Layer

**Figure 2:**
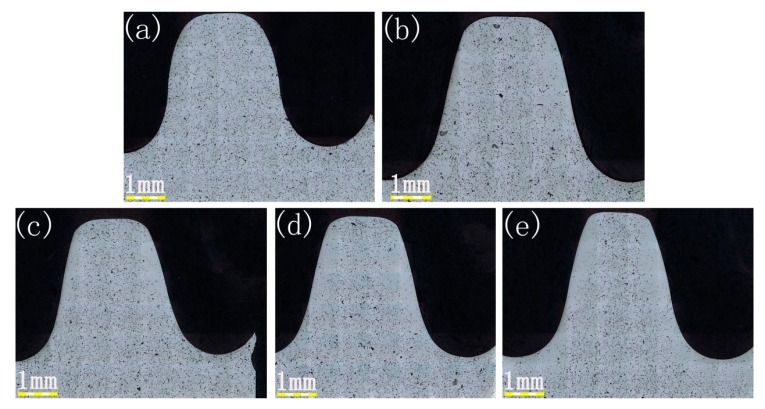
Metallographs of the tooth of the sprocket with different rolling feeds. (**a**) △d = 0 mm; (**b**) △d = 0.2 mm; (**c**) △d = 0.4 mm; (**d**) △d = 0.6 mm; and (**e**) △d = 0.8 mm.

**Figure 3:**
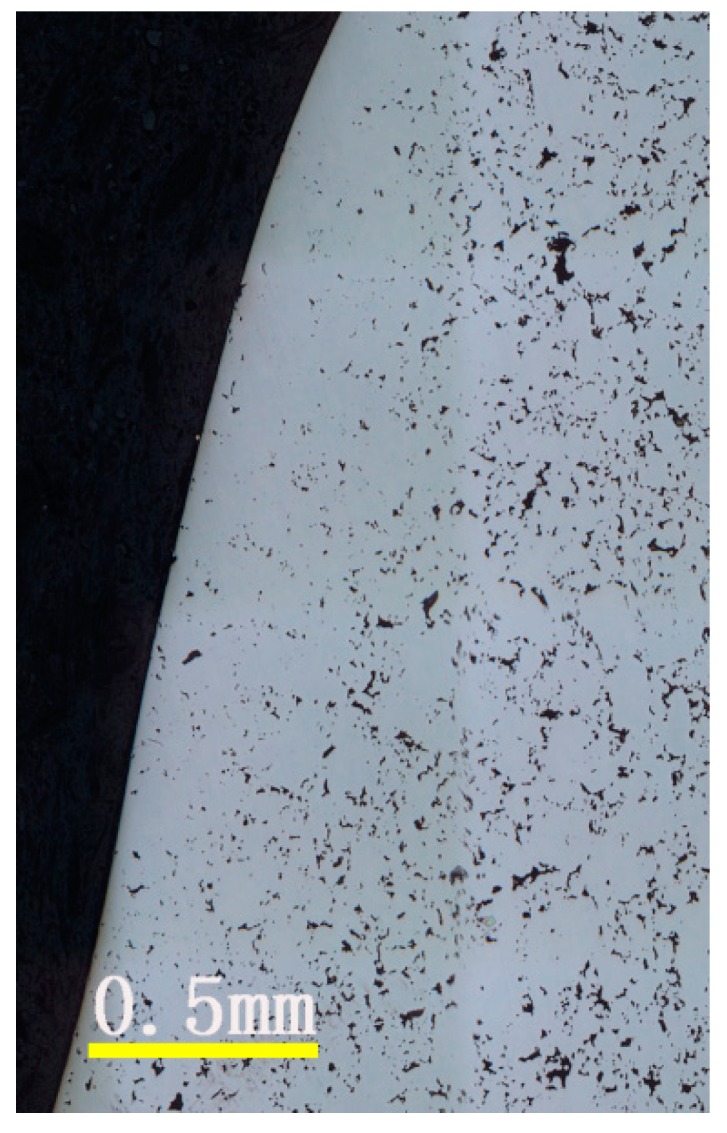
Enlarged view of the metallograph of the tooth of the sprocket after the rolling.

Cross-section metallographs of a sprocket with different rolling feeds were shown in Figure 2. It can be seen that the sprocket had many pores in both the surface and core layer before the rolling process. However, after the rolling process, a densified region occurred and the depth of that region was gradually increased with the increase of the rolling feed, as shown in Table 2. Figure 3 is an enlarged view of the metallograph of the surface after rolling the part. From the figure, we can see there are still many pores in the core part but a homogenous densified layer on the surface. In the rolling process, the rolling dies create a force perpendicular to the surface, which led to the plastic deformation of the sprocket and the surface of the component flowed with the force, which can reduce or even eliminate the pores in the surface region. Therefore, we can obtain a densified layer through the rolling process.

**Figure 4:**
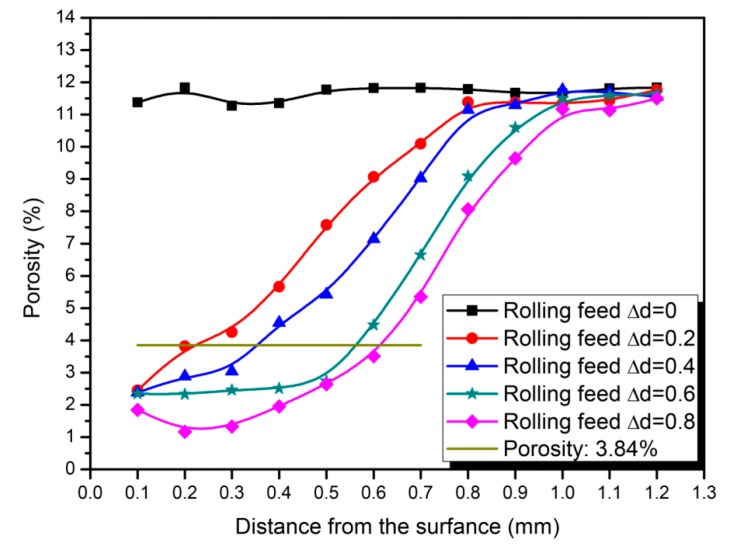
Comparison of porosity distributions with different rolling feeds.

Figure 4 shows a comparison of the porosity distributions from the surface layer to the central part. It can been seen from the picture that the porosity distributions of the surface layer and the central part were almost the same (about 9%–12%). After the rolling process, the porosity of the surface layer decreased. The decrease of the porosity was inversely proportional to the distance from the surface. After the rolling process, the surface layer almost reached a densified state, while the core portion was almost substantially unchanged.

For the Fe-base PM components, the density is 7.8 g/cm^3^ when reaching the fully-densified state, and it can be considered as a densified state when the porosity is decreased below 3.84% and the density reaches 7.5 g/cm^3^. It can be concluded from Figure 2 and Figure 4 that, with the increasing rolling feed, the depth of the densified layer increased. When the rolling feed △d reaches 0.8 mm, the depth of the densified layer reaches 0.61 mm. After the rolling feed reaches 0.6, the increase of the depth of the densified layer slows down. This is mainly due to the work hardening effect which can impede the plastic deformation.

**Figure 5:**
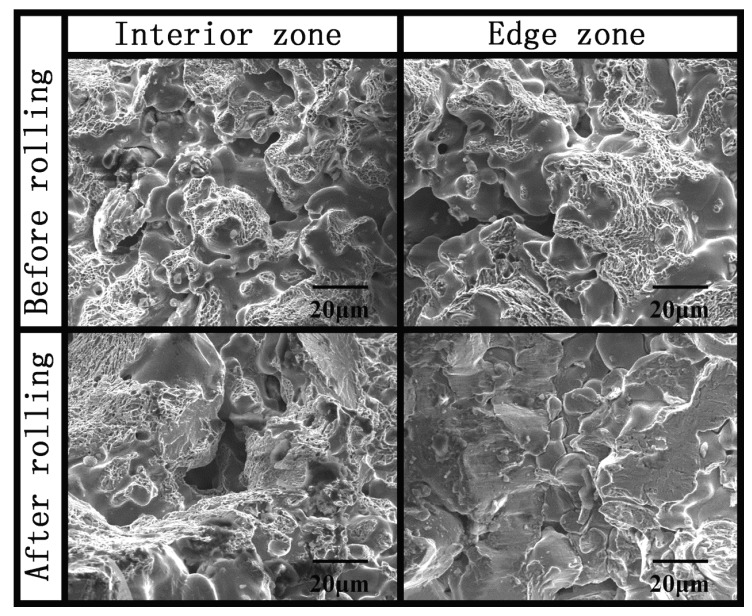
SEM images of interior zones and edge zones of the pre-rolled and post-rolled part.

SEM images of the internal and edge regions of the fracture were taken for both the pre-rolled and post-rolled gears, as is shown in Figure 5. For the pre-rolled sprocket, both the internal and edge regions showed typical dimple fracture morphologies which indicate the ductile fracture. After the rolling process, trans crystalline fracture occurred instead of the ductile fracture in the edge. This means edge strength was significantly improved through rolling. 

### 3.2. Micro-Hardness Analysis

**Figure 6:**
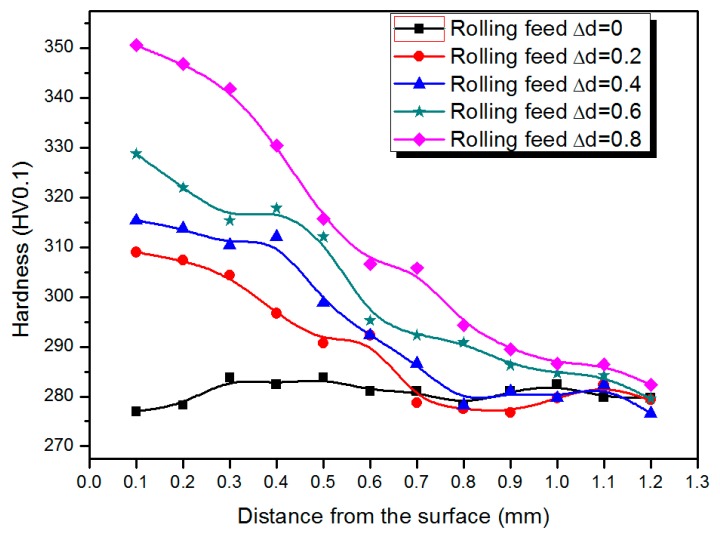
Micro-hardness distributions of gears with different rolling feeds.

Figure 6 shows a comparison of the micro-hardness with different rolling feeds. Micro-hardness distribution curves of samples of different rolling feed show the surface hardness of the sample significantly increased after rolling, but there was barely any change in the core region. This also confirmed that the surface plastic deformation caused by rolling can effectively improve the surface hardness of PM components. As the rolling feed increased from 0.2 to 0.8 mm, the hardness of the surface also increased form 309 HV to 327 HV. In the edge portions, the hardness values have a stable platform, the platform width decreases with the increasing rolling feed. For PM components, density has a direct impact on the mechanical properties of the material. Thus, the sprocket with the largest rolling feed (0.8 mm) should have the largest surface density since it had the highest hardness (Figure 6).

### 3.3. Roughness Analysis

**Figure 7:**
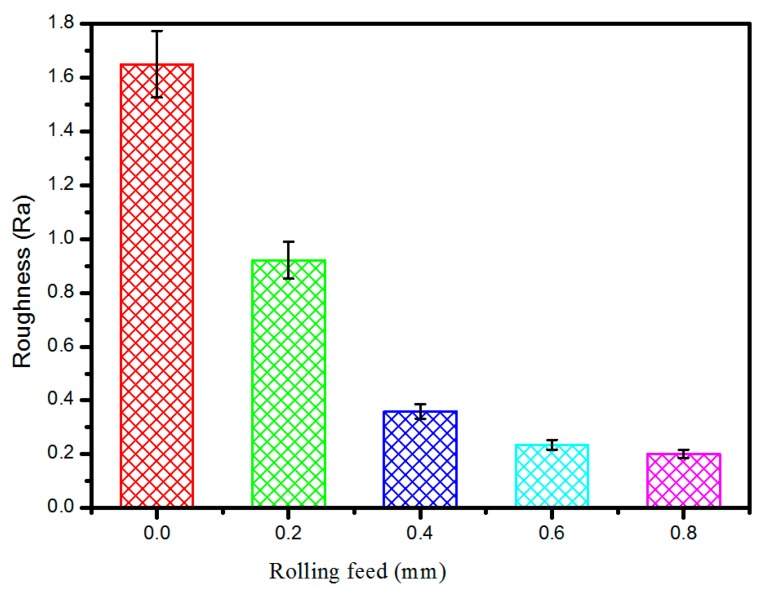
Comparison of tooth flank roughness.

Figure 7 shows the comparison of tooth flank roughness with different rolling feeds. It can be seen that the tooth flank roughness decreased with the increasing rolling feed, which can be explained by two aspects. First, with the increased amount of the feed roller, the porosity of the tooth flank decreased, which can increase the density and reduce the impact of pores on the gap roughness. Second, as the feed rate increases the higher pressure, along with the increased rolling feed, plastic deformation occurred more significantly to eliminate the projecting part while the concave parts were barely affected. Thus, with the increased rolling feed, tooth flank roughness is reduced.

### 3.4. Wear Resistance Analysis

It can be concluded from the above data that through the rolling process, the density and hardness of the gears can be improved, while the roughness can be reduced. As is well known, the wear resistance increases with the density. This is mainly due to the larger effective contact area and roughness of the components. Thus, the wear resistance should be improved with the rolling process.

To improve the wear resistance, cross-section nano-scratch tests were carried out on the pre-rolled and post-rolled gears. Nano-scratch tests were set at a fixed carrier mode, and the scoring load was 10 mN. Curvature radius of the diamond ball tip was 2 μm, the scribing speed was 0.6 mm/min, and the scratch length was 1.5 mm. The scoring process was divided into three states. During the first state, a pre-scanning using a scanning load of 0.5 mN was applied to obtain the changes in displacement of the material surface morphology. During the second stage, force-displacement curves were obtained using the scoring load, and the frictional force-displacement relationship was then calculated. The scanning load was used to measure the change in residual scratch depth with displacement in the final stage.

**Figure 8:**
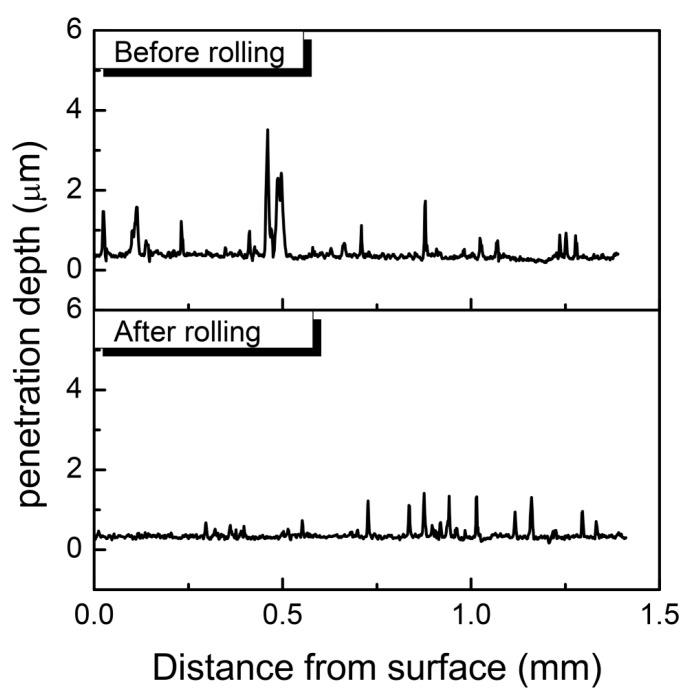
Penetration depth of pre-rolled and post-rolled gears.

Enhancement of wear resistance through rolling processes can be proved by the nano-scratch test. The curves of the penetration depth and length are shown in Figure 8. As can be seen from the figures, the curve of the pre-rolling sprocket had some high peaks in the near surface position (in the range of 0–0.5 mm), while the post-rolled sprocket curve was quite smooth. This should owe to the disorder distribution of pores in the sprocket before rolling, while the surface layer of the post-rolled sprocket was densified. The average penetration depth was 0.37 μm and 0.29 μm for the pre-rolled and post-rolled gears, respectively. This means that with the increasing amount of feed, hardness and resistance to deformation of the gears can be increased, and the wear resistance can also be improved.

**Figure 9:**
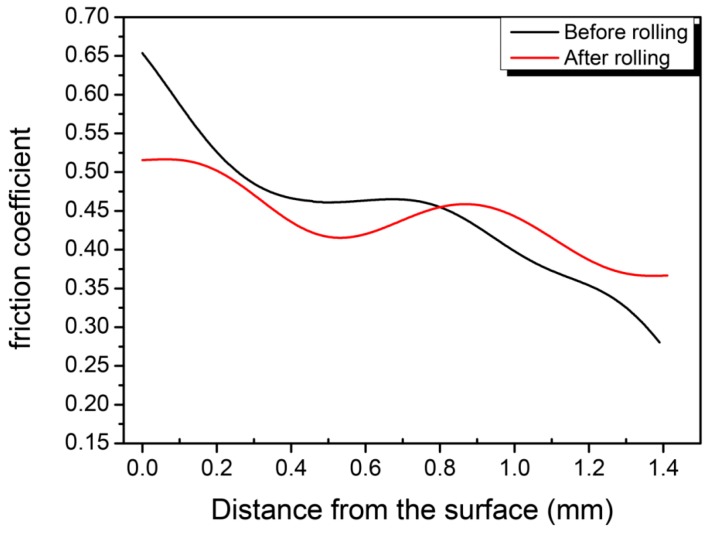
Friction coefficient curve of pre-rolled and post-rolled gears.

Figure 9 shows the curve of the friction coefficient with scribing length in pre-rolled and post-rolled sprockets. As can be seen from the figure, the friction coefficient can be divided into three stages. For the pre-rolled sample, the friction coefficient kept decreasing with the increasing scribing length. In the beginning, the coefficient of friction rapidly decreases from a maximum value of 0.65. During the second stage, with the scribing length of 0.3–0.8 mm, the friction coefficient was constantly around 0.47. During the third stage, after reaching 0.8 mm, the friction coefficient decreased to 0.28 with a decreasing rate slightly lower than that in the first stage. The friction coefficient of the post-rolled samples decreased from the maximum 0.51 to 0.41 in the first stage and slightly increased to 0.45 during the second stage. In the third stage (the scribing length is over 0.9 mm), the friction coefficient rapidly reduced.

According to the adhesion-furrow friction theory [18], the reduction of the friction coefficient in the first state should be attributed to the use of a constant loading mode, which results in a small contact area between the diamond indenter and the sample at the beginning stage and the friction mechanism can be considered as an adhesion mode. When the friction proceeds, the contact area between the indenter and the sample increases, which leads to a furrow effect in the mechanism of friction and the adhesion effect becomes weak. Therefore, the friction coefficient decreased rapidly. For rolled gears, in the first stage, there is a stable platform in the friction coefficient curve. This was due to the uniform densified layer caused by rolling. The width of the platform reflects the depth of the surface densified layer. The increased surface density is effective in improving the adhesive-type wear resistance. It is known that pores in the PM gears may cause stress concentration and the cracks always occurs around the pores [19]. Cracks can spread through the pores and cause worse fracture. Thus, the densified layer can offer better wear resistance and higher strength for the PM gears.

## 4. Conclusions

A uniform densified layer can be achieved through surface rolling processes, and mechanical properties of PM components can be improved. With an increase of the rolling feed, the depth of the densified layer increased and, thus, can improve the mechanical properties, such as hardness and wear resistance, and decrease the surface roughness. The improvement of the mechanical properties in the PM gears can expand their application area in the auto and other industries.

## Figures and Tables

**Table 1:** Chemical composition of the PM material.

**Element**	Cr	C	Cu	Astaloy 85Mo
**Content (%)**	0.85	0.80	1.50	Bal.

**Table 2:** Depth of the densified layer with different rolling feeds.

**Rolling Feed △d (mm)**	0	0.2	0.4	0.6	0.8
**Depth of Densification Layer (mm)**	0	0.16	0.33	0.56	0.61
